# The role of REST and HDAC2 in epigenetic dysregulation of Nav1.5 and nNav1.5 expression in breast cancer

**DOI:** 10.1186/s12935-017-0442-6

**Published:** 2017-08-01

**Authors:** Nur Sabrina Kamarulzaman, Hemaniswarri Dewi Dewadas, Chiuan Yee Leow, Nik Soriani Yaacob, Noor Fatmawati Mokhtar

**Affiliations:** 1 Institute for Research in Molecular Medicine (INFORMM), Universiti Sains Malaysia, Health Campus, 16150 Kubang Kerian, Kelantan Malaysia; 2 Department of Chemical Pathology, School of Medical Sciences, Universiti Sains Malaysia, Health Campus, 16150 Kubang Kerian, Kelantan Malaysia

**Keywords:** Nav1.5, nNav1.5, REST, HDAC2, TSA, Breast cancer, Aggressiveness, MMP2, N-cadherin

## Abstract

**Background:**

Increased expression of voltage-gated sodium channels (VGSCs) have been implicated with strong metastatic potential of human breast cancer in vitro and in vivo where the main culprits are cardiac isoform Nav1.5 and its ‘neonatal’ splice variant, nNav1.5. Several factors have been associated with Nav1.5 and nNav1.5 gain of expression in breast cancer mainly hormones, and growth factors.

**Aim:**

This study aimed to investigate the role of epigenetics via transcription repressor, repressor element silencing transcription factor (REST) and histone deacetylases (HDACs) in enhancing Nav1.5 and nNav1.5 expression in human breast cancer by assessing the effect of HDAC inhibitor, trichostatin A (TSA).

**Methods:**

The less aggressive human breast cancer cell line, MCF-7 cells which lack Nav1.5 and nNav1.5 expression was treated with TSA at a concentration range 10–10,000 ng/ml for 24 h whilst the aggressive MDA-MB-231 cells was used as control. The effect of TSA on Nav1.5, nNav1.5, REST, HDAC1, HDAC2, HDAC3, MMP2 and N-cadherin gene expression level was analysed by real-time PCR. Cell growth (MTT assay) and metastatic behaviors (lateral motility and migration assays) were also measured.

**Results:**

mRNA expression level of Nav1.5 and nNav1.5 were initially very low in MCF-7 compared to MDA-MB-231 cells. Inversely, mRNA expression level of REST, HDAC1, HDAC2, and HDAC3 were all greater in MCF-7 compared to MDA-MB-231 cells. Treatment with TSA significantly increased the mRNA expression level of Nav1.5 and nNav1.5 in MCF-7 cells. On the contrary, TSA significantly reduced the mRNA expression level of REST and HDAC2 in this cell line. Remarkably, despite cell growth inhibition by TSA, motility and migration of MCF-7 cells were enhanced after TSA treatment, confirmed with the up-regulation of metastatic markers, MMP2 and N-cadherin.

**Conclusions:**

This study identified epigenetics as another factor that regulate the expression level of Nav1.5 and nNav1.5 in breast cancer where REST and HDAC2 play important role as epigenetic regulators that when lacking enhances the expression of Nav1.5 and nNav1.5 thus promotes motility and migration of breast cancer. Elucidation of the regulatory mechanisms for gain of Nav1.5 and nNav1.5 expression may be helpful for seeking effective strategies for the management of metastatic diseases.

## Background

The ‘classic’ role of voltage-gated sodium channels (VGSC) is to mediate cell regenerative membrane depolarization and conduction of electrical signaling (action potentials) in traditionally ‘excitable’ cells such as nerve, skeletal, and heart muscle cells [1]. Recent evidences show that VGSCs are also functionally expressed in many types of carcinomas (cancers of epithelial origin), including those of prostate [2, 3], breast [4, 5], lung (small-cell, non-small-cell and mesothelioma) [6–8], cervix [9], ovary [10], and colon [11].

In breast cancer, increased VGSC expression and activity, predominantly the cardiac isoform Nav1.5 and its ‘neonatal’ splice variant, nNav1.5, correlates positively with metastatic potential in vitro [5]. In vivo, the mRNA expression of Nav1.5 and nNav1.5 was detected in biopsy samples of breast cancer with occurrence of lymph node metastasis [5] and in breast tumors [12]. Ex vivo, using whole-cell patch clamp, cells in breast tumor tissue slices displayed fast inward Na^+^ currents similar to currents detected in the aggressive human breast cancer cell line, MDA-MB-231 [13].

Apparently, a full elucidation of the regulatory mechanisms for such gain of expression of Nav1.5 and nNav1.5 may be helpful for seeking effective strategies for the management of metastatic diseases. At present, various studies have demonstrated that growth factors (epidermal growth factor, nerve growth factor) [14, 15], hormones [16], Na^+^ concentration [17], and VGSC auxiliary β-subunits [18] can all regulate the expression of VGSC in cancer cells thus potentiating aggressiveness. Recently, epigenetic changes has been suggested to contribute to the overexpression of VGSC in aggressive cancer cells [19, 20], though the notion remains to be tested. Herein, the role of epigenetics via transcription repressor, repressor element silencing transcription factor (REST) and histone deacetylases (HDACs) in enhancing Nav1.5 and nNav1.5 expression in human breast cancer cells was assessed.

In classic non-excitable cells i.e. epithelial cell, the expression of a number of genes commonly expressed in neurons including VGSC e.g. Nav1.2 is repressed by the master of transcription repressor, REST [21–23]. REST is a zinc finger protein that binds to a conserved repressor element-1 in a large number of genes encoding fundamental neuronal traits [23] such as ion channels, synaptic vesicle proteins, and neurotransmitter receptors. REST mediates active repression via recruitment of HDACs [24, 25]. With the emergence of neuronal-like adaptation of cancer to gain aggressiveness [26], REST has now proven to play important tumor-suppressor function [27].

HDAC is a chromatin modifying enzyme involved in the removal of an acetyl group on lysine residues of histone protein contributing to transcriptional silencing of genes [28]. So far, eighteen HDAC isoforms, grouped into four classes have been described in humans [29]. Owing to the discovery of epigenetic drugs such as HDAC inhibitors, cellular functions of HDAC on gene expression is progressively deciphered. Of particular, these drugs have led to better understanding for the role of HDAC in cancers. The best characterised and probably biologically most relevant HDAC in human cancers are class I isoforms HDAC1, HDAC2, and HDAC3 [30]. The expression patterns of HDAC1, HDAC2, and HDAC3 have been evaluated in different types of cancers, including gastric cancer [31], liver cancer [32], prostate cancer [33], breast cancer [34], renal cell cancer [35], ovarian [36] and endometrial carcinomas [37].

This study focused on examining the role of REST, HDAC1, HDAC2, and HDAC3 on influencing the expression of Nav1.5 and nNav1.5 in breast cancer that promote aggressiveness. In doing so, the expression level of Nav1.5, nNav1.5, REST, HDAC1, HDAC2, and HDAC3 were measured and compared between the less aggressive human breast cancer cells, MCF-7 and the aggressive, MDA-MB-231 cells. The effect of HDAC inhibitor, trichostatin A (TSA), on Nav1.5, nNav1.5, HDAC1, HDAC2, HDAC3, REST, MMP2 and N-cadherin gene expression and on cell motility and migration of the less metastatic human breast cancer cells, MCF-7, were investigated.

## Methods

### Cell culture

MCF-7 and MDA-MB-231 cell line were purchased from the American Type Culture Collection (ATCC) and were cultured in 10 cm or 35 mm plastic culture dishes (Becton–Dickinson, USA). Both of the cells were maintained in Dulbecco’s minimum essential medium (DMEM) (Nacalai Tesque, Japan) supplemented with 4 mM l-glutamine (Gibco, USA), 5% of fetal bovine serum (FBS) (Gibco, USA) and 1% penicillin–streptomycin (Gibco, USA). Cultured cells were kept in a humidified incubator at 37 °C, 100% relative humidity and with 5% regulated CO_2_.

### Chemicals

TSA was purchased from Invivogen, USA and stock solutions were prepared in 100% ethanol (Nacalai Tesque, Japan), sterile filtered and stored at −20 °C until use.

### Cell viability and proliferation assays

Briefly, 3 × 10^4^ cells/well were cultured in a 96-well culture plate and incubated at 37 °C for 24 h. The cells were treated with different concentrations of TSA for 24, 48 and 72 h in triplicates. Medium was removed and 100 μl of DMEM and 10 μl of 12 mM 3-[4,5-dimethylthiazol-2-yl]-2,5-diphenyltetrazolium bromide (MTT) (Invitrogen, USA) were added to each well and incubated for 4 h. 85 μl of MTT solution was removed followed by addition of 50 μl dimethyl sulfoxide (DMSO) (Fisher Scientific, USA). The plate was incubated at 37 °C for 10 min and the absorbance was measured at 540 nm using SpectraMax M5 microplate reader (Molecular Device, USA).

### Cell treatment regimes

5 × 10^5^ cells were cultured in 35 mm culture dish and were incubated at 37 °C for 24 h. Media was removed and TSA was added to the cells following the treatment concentration. The cells were incubated for 24 h and preceded for RNA extraction.

### Quantitative real-time reverse transcription polymerase chain reaction (qRT-PCR) analysis

Total RNA was extracted using Sepasol method according to the protocol provided by the manufacturer (Nacalai, Tesque, Japan). Total RNA (1 µg) was converted to cDNA using the QuantiTect Reverse Transcription Kit (Qiagen, Germany) according to the manufacturer’s protocol. Real-time PCR was performed using SensiFAST SYBR Hi-ROX kit (Bioline, UK) according to manufacturer’s protocol, in triplicates. Sequence primers used were as follows: β-actin forward, ATTGCCGACAGGATGCAGAAG-3′ and reverse, 5′-AGAAGCATTTGCGGTGGACG-3′. Nav1.5 forward, 5′-TTGCTTGTTATGGTCATTGGC-3′ and reverse, 5′-GTTGTTCATCTCTCTGTCCTCAT-3′, nNav1.5 forward, 5′-CTGCACGCGTTCACTTTCCT-3′ and reverse, 5′-GACAAATTGCCTAGTTTTATATTT-3′, HDAC1 forward, 5′-GGAAATCTATCGCCCTCACA-3′ and reverse, 5′-TTGCCACAGAACCACCAGTA-3′, HDAC2 forward, 5′-CCGTCTACCATGATGATCCTG-3′ and reverse 5′-GTCTTCACTGTTCCATCTCCTC-3′, HDAC3 forward, 5′-CGCTTCCACTCCGAGGACTA-3′ and reverse, 5′-CTGGGCAGTCATCGCCTAC-3′, REST forward, 5′-ACTAGACATATGCGTACTCATTCAG-3′ and reverse 5′-CCATTGTGAACCTGTCTTGC-3′, MMP2 forward, 5′-TCACTCCTGAGATCTGCAAACAG-3′ and reverse 5′-TCACAGTCCGCCAAATGAAC-3′, and N-cadherin forward, 5′-GTCAACATCACTCTCACCGA-3′ and reverse 5′-ATCCACAGCTCTTATTCTTCCA-3′. Quantitative real-time was performed in an ABI Prism 7000 Sequence Detection System (Life Technologies, USA) and the amplification conditions were as follows: initial activation for 10 min at 95 °C for one cycle, 10 s at 95 °C and 30 s at 60 °C for 34 cycles. Ct values of target genes were normalised to β-actin and the relative mRNA expression of target genes were calculated by the 2^−ΔΔCt^ method [38].

### Wound healing assay

Lateral motility of cells was assessed using a monolayer wound healing assay. Cells were plated in 35 mm plastic culture dish at an initial density of 5 × 10^5^ cells/dish and allowed to settle for 24 h prior to wound creation and any pharmacological treatment (T_0_). Wounds were created by scratching the monolayer c ells using a p200 pipette tip at 90° angle. Wound widths were measured again after 24 h (T_24_). Lateral motility was calculated according to the motility index formula 1 − (T_24_/T_0_) [39] and the percentage of lateral motility was normalised to control (untreated).

### Migration assay

Migration assay was performed using transwell chambers with 8 μm pore inserts (Corning, USA). Briefly, 5 × 10^4^ cells in 300 μl of 1% serum medium were plated into the upper chambers and 750 μl medium with 10% serum was added as chemo attractant in lower chambers. After 24 h incubation, non-migrating cells were gently removed using cotton swab and the migrated cells (at the outer surface of membrane insert) were fixed with ice-cold methanol for 15 min. The cells were stained in 0.05% crystal violet for 15 min. Migrated cells were viewed under inverted microscope (10× magnification) and images were captured using a monochrome ProgRes CF^cool^ CCD camera (Jenoptik, Germany). Migrated cells in each insert were counted and averaged from 30 random fields.

### Statistical analysis

All data are expressed as the mean ± SEM. Student’s *t* test was carried out to evaluate differences between two groups (treated vs untreated). Differences were considered to be significant for values of p < 0.05.

## Results

### MCF-7 cells expressed low level of Nav1.5 and nNav1.5 but higher REST expression

**Fig. 1:**
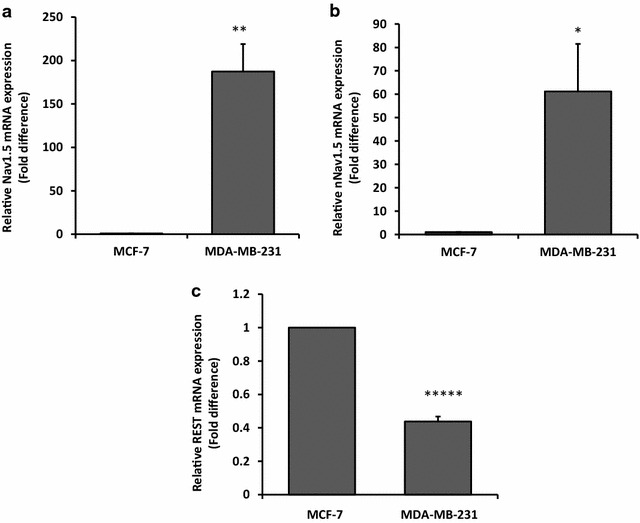
MCF-7 express low expression of Nav1.5 and nNav1.5 but higher REST expression compared to MDA-MB-231. Relative mRNA expression level of Nav1.5 and nNav1.5 was measured using qRT-PCR where β-actin was used as housekeeping gene. **a** The expression of Nav1.5 in MDA-MB-231 normalised to MCF-7 cells. **b** The expression of nNav1.5 in MDA-MB-231 normalised to MCF-7 cells. **c** The expression of REST in MDA-MB-231 normalised to MCF-7 cells. Data were collected from n = 3 independent experiments, presented as mean ± SEM. Unpaired Student’s *t* test *p < 0.05, **p < 0.01, and *****p < 0.00001

We compared the gene expression level of Nav1.5, nNav1.5 and REST by qRT-PCR in two human breast cancer cell lines, MDA-MB-231 (the highly aggressive human breast cancer cells) and MCF-7 (the less aggressive human breast cancer cells). The expression level of Nav1.5 and nNav1.5 was very low in MCF-7 cells compared to MDA-MB-231 cells. MDA-MB-231 cells expressed 187 ± 31.5-fold (p < 0.01) and 61 ± 20.4-fold (p < 0.05) greater Nav1.5 and nNav1.5 mRNA expression, respectively, compared to MCF-7 cells (Fig. 1a, b). The expression level of REST was significantly lower in MDA-MB-231 cells (0.4 ± 0.03-fold, p < 0.00001) compared to MCF-7 cells.

### MDA-MB-231 cells expressed low level of HDAC1, HDAC2, and HDAC3

**Fig. 2:**
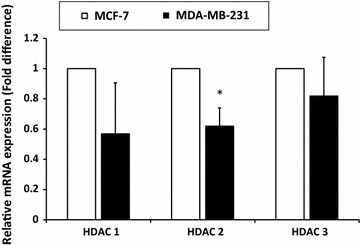
HDAC2 is significantly lower in MDA-MB-231 cells compared to MCF-7 cells. Relative mRNA expression level of HDAC1, HDAC2 and HDAC3 was measured using qRT-PCR where β-actin was used as housekeeping gene. mRNA expression of each HDAC in MDA-MB-231 was normalised to HDAC in MCF-7 cells. Data were collected from n = 3 independent experiments, presented as mean ± SEM. Unpaired Student’s *t* test *p < 0.05

We measured the basal expression levels of HDAC1, HDAC2 and HDAC3 in MDA-MB-231 cells compared to MCF-7 cells (without TSA treatment). As presented in Fig. 2, HDAC1, HDAC2 and HDAC3 exhibited lower mRNA expression in MDA-MB-231 compared to MCF-7 cells. However, only HDAC2 showed a significantly lower expression in MDA-MB-231 cells (p < 0.05).

### TSA increased the mRNA expression level of Nav1.5 and nNav1.5 in MCF-7 cells

**Fig. 3:**
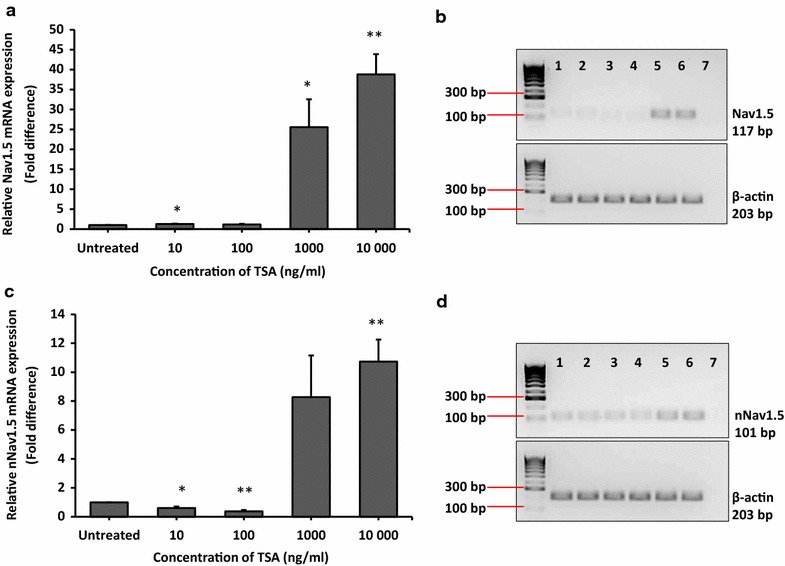
TSA increased the expression of Nav1.5 and nNav1.5 in MCF-7 cells. MCF-7 cells were treated with 10–10,000 ng/ml TSA for 24 h. Relative mRNA expression level was measured using qRT-PCR where β-actin was used as housekeeping gene. **a** Relative mRNA expression level of Nav1.5 normalised to untreated in MCF-7 cells after treatment. **b** Gel electrophoresis images of qRT-PCR products of Nav1.5. **c** Relative mRNA expression level of nNav1.5 normalised to untreated in MCF-7 after treatment. **d** Gel electrophoresis images of qRT-PCR products of Nav1.5. For gel images, *lane 1* untreated, *lane 2* control ethanol (qRT-PCR data not shown), *lanes 3–6* 10, 100, 1000 and 10,000 ng/ml TSA, *lane 7* non-template control. Data were collected from n = 3 independent experiments, presented as mean ± SEM. Unpaired Student’s *t* test *p < 0.05 and **p < 0.01

Next, we examined the effect of TSA treatment on Nav1.5 and nNav1.5 mRNA expression by qRT-PCR. In comparison to untreated cells, our results showed that treatment with 1000 and 10,000 ng/ml TSA for 24 h significantly increased the expression of Nav1.5 by 26 ± 7.0-fold (p < 0.05) and 39 ± 5.1-fold (p < 0.01), respectively (Fig. 3a and b). Similarly, the expression of nNav1.5 was increased by 8 ± 2.9-fold and 11 ± 1.5-fold (p < 0.01) with 1000 and 10,000 ng/ml TSA, respectively (Fig. 3c and d).

### TSA reduced the mRNA expression level of REST in MCF-7 cells

**Fig. 4:**
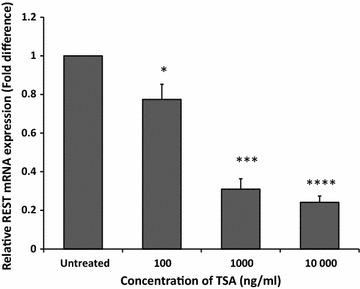
TSA downregulated REST gene expression in MCF-7 cells. MCF-7 cells were treated with 10–10,000 ng/ml TSA for 24 h. Relative mRNA expression level of REST was measured using qRT-PCR where β-actin was used as housekeeping gene and normalised to untreated. Data were collected from n = 3 independent experiments, presented as mean ± SEM. Unpaired Student’s *t* test *p < 0.05, ***p < 0.001 and ****p < 0.0001

In comparison to untreated cells, our results showed that gene expression of REST was significantly down-regulated to 0.8 ± 0.1 (p < 0.05), 0.3 ± 0.1 (p < 0.001) and 0.2 ± 0.03 (p < 0.0001) with increasing dose of 100, 1000 and 10,000 ng/ml TSA, respectively (Fig. 4).

### HDAC2 mRNA expression level was downregulated by TSA in MCF-7 cells

**Fig. 5:**
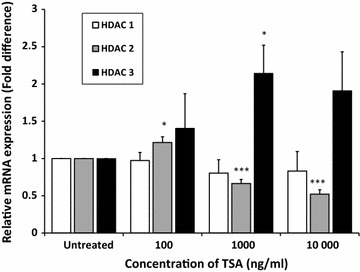
TSA reduced the HDAC1 and HDAC2 expression. MCF-7 cells were treated with 10–10,000 ng/ml TSA for 24 h. Relative mRNA expression level of HDAC1, HDAC2 and HDAC3 was measured using qRT-PCR where β-actin was used as housekeeping gene and normalised to untreated. Data were collected from n = 4 independent experiments, presented as mean ± SEM. Unpaired Student’s *t* test *p < 0.05 and ***p < 0.001

Next, the effect of TSA on the mRNA expression of HDAC1, HDAC2 and HDAC3 in MCF-7 cells were analysed by qRT-PCR. The expression of HDAC1 was reduced but not significant, with concentrations of 100–10,000 ng/ml TSA by 0.97 ± 0.1-fold, 0.8 ± 0.2-fold and 0.83 ± 0.3-fold, respectively. HDAC2 expression was significantly down-regulated by ~0.6 ± 0.1-fold (p < 0.001) and ~0.5 ± 0.1-fold (p < 0.001) with concentrations of 1000 and 10,000 ng/ml TSA, respectively. In contrast, the expression of HDAC3 was significantly increased by 2 ± 0.4-fold (p < 0.05) with 1000 ng/ml TSA (Fig. 5).

### TSA caused growth suppression in MCF-7 cells

**Fig. 6:**
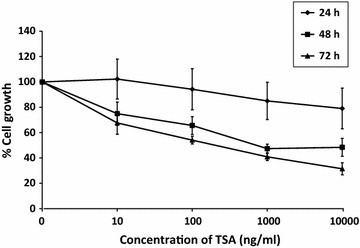
TSA caused dose- and time-dependent growth suppression in MCF-7 cells. MCF-7 cells were treated with TSA at concentration of 10–10,000 ng/ml for 24–72 h and cell growth was measured using MTT assay. The cell growth of untreated control cells was considered as 100%. Data were collected from n = 3 independent experiments, presented as mean ± SEM

To determine the effect of TSA on cell growth, MCF-7 cells were treated with 10–10,000 ng/ml of TSA for 24, 48 and 72 h. As presented in Fig. 6, cell growth was reduced in dose- and time-dependent manner by TSA. Cell growth at 24, 48, and 72 h was reduced by 21 ± 16.0, 52 ± 7 and 69 ± 4.7%, respectively, with 10,000 ng/ml TSA.

### TSA enhanced lateral motility and migration of MCF-7 cells

**Fig. 7:**
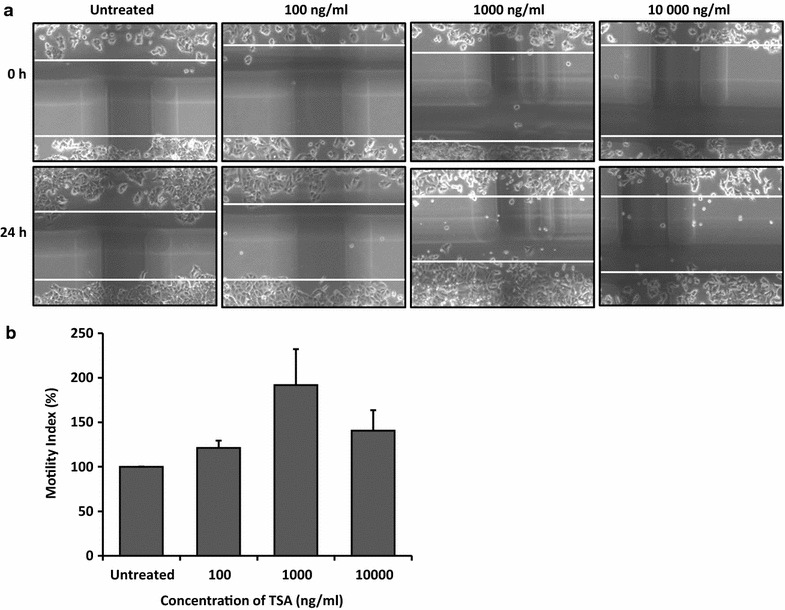
TSA enhanced motility of MCF-7 cells. Lateral motility was assed according to wound healing assay. **a** (*upper panel*) Representative scratch wound images of MCF-7 cells at 0 h and (*lower panel*) 24 h treated with TSA at 100–10,000 ng/ml. **b** The percentage of motility index was quantified according to the formula 1 − (T_24_/T_0_) and normalised to untreated. Data were collected from n = 3 independent experiments, presented as mean ± SEM

**Fig. 8:**
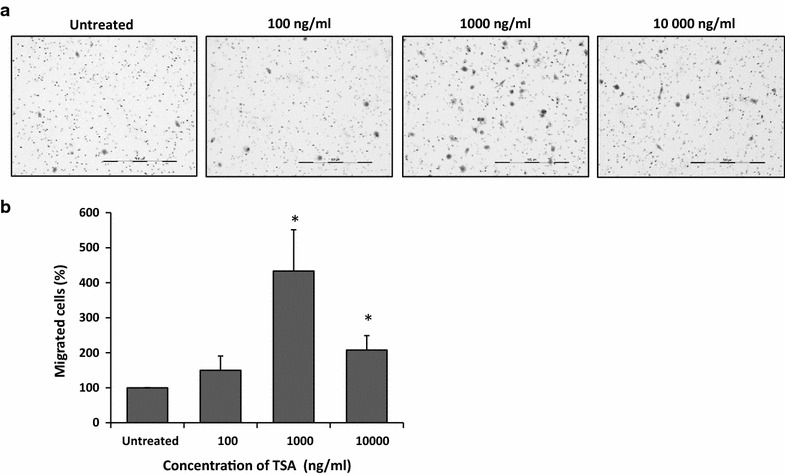
TSA enhanced migration of MCF-7 cells. Migration was assessed according to transwell migration assay. Migrated cells were viewed under inverted microscope (×10 magnification) and images were captured using a monochrome ProgRes CF^cool^ CCD camera (Jenoptik, Germany). Migrated cells in each insert were counted and averaged from 30 random fields. **a** Representative images of migrated MCF-7 cells from one field view. **b** The percentage of migrated cells normalised to untreated. Data were collected from n = 3 independent experiments, presented as mean ± SEM. Unpaired Student’s *t* test *p < 0.05

To investigate the effect of TSA on cell metastatic behaviors of MCF-7 cells, we performed wound healing and migration assay. As shown in Fig. 7, cell motility was enhanced by ~20 ± 8.2% and ~90 ± 40.2% at concentration of 100 and 1000 ng/ml TSA, respectively. However, at 10,000 ng/ml TSA, motility was only enhanced by 40 ± 23.0%, compared to the untreated cells due to toxicity effect. Similarly, our data revealed that TSA at 1000 ng/ml significantly enhanced the migration of MCF-7 cells by ~300 ± 117.5% (p < 0.05) (Fig. 8). Again, at 10,000 ng/ml TSA, migration was only enhanced by 107 ± 41.1% (p < 0.05), compared to the untreated cells due to growth inhibition effect.

### mRNA expression of MMP-2 and N-cadherin was upregulated by TSA

**Fig. 9:**
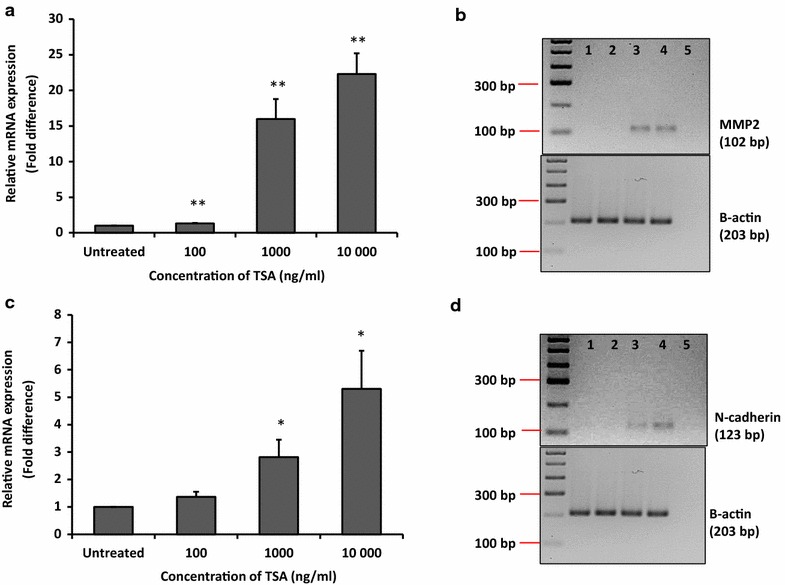
MMP2 and N-cadherin mRNA expression in MCF-7 cells was upregulated by TSA. Relative mRNA expression level was measured using qRT-PCR where β-actin was used as housekeeping gene. **a** Relative mRNA expression level of MMP2 normalised to untreated. **b** Gel electrophoresis images of qRT-PCR products of MMP2. **c** Relative mRNA expression level of N-cadherin normalised to untreated. **d** Gel electrophoresis images of qRT-PCR products of N-cadherin. For gel images, *lane 1* untreated, *lanes 2–4* 100, 1000 and 10,000 ng/ml TSA, *lane 5* non-template control. Data were collected from n = 3 independent experiments, presented as mean ± SEM. Unpaired Student’s *t* test *p < 0.05 and **p < 0.01

The mRNA expression of two metastasis markers, MMP2 and N-cadherin was investigated to confirm increased in metastatic behaviours in MCF-7 cells by TSA. We found that mRNA expression of MMP2 was significantly upregulated by ~16 ± 2.8-fold (p < 0.01) and 22 ± 2.9-fold (p < 0.01) with concentrations of 1000 and 10,000 ng/ml TSA, respectively (Fig. 9a and b). Similarly, N-cadherin mRNA expression was also enhanced by 2.8 ± 0.6-fold (p < 0.05) and 5.3 ± 1.4-fold (p < 0.05) at the same concentrations of TSA where MMP2 mRNA expression increased (Fig. 9c and d).

## Discussion

In search to understand factors that contribute to the enhancement of VGSC expression in aggressive cancers, reports have demonstrated that the primary regulators are hormones [16], growth factors [14, 15], auxiliary β-subunits [18] and auto-regulation via Na^+^ concentration [17, 38]. Though studies have shown that VGSCs are highly expressed in various tumour types with no alteration of their genetic code [19, 20], epigenetic regulation of VGSCs in these cancers is not known.

To our knowledge, this is the first investigation on the regulatory role of epigenetic regulators, REST and HDACs on the expression of Nav1.5 and nNav1.5 in breast cancer that promote aggressiveness.

Firstly, we showed that less aggressive human breast cancer cells, MCF-7 expressed significantly lower level of Nav1.5 (p < 0.01) and nNav1.5 (p < 0.05) mRNA expression than aggressive human breast cancer cells, MDA-MB-231. Inversely, basal mRNA expression level of REST, HDAC1, HDAC2 and HDAC3 was higher in MCF-7 compared to MDA-MB-231 cells. In excitable cells e.g. neurons, REST dependence of VGSC expression suppression has been reported and confirmed several time [22, 40–42]. However in cancers, increased expression of Nav1.5/nNav1.5 and lack of REST expression level have been separately reported to be associated with breast cancer aggressive phenotype [5, 39, 40, 43]. Meanwhile, in adult heart, inhibition of REST resulted in re-expression of various neonatal genes including those encoding ‘neonatal’ ion channels such as the hyperpolarization-activated, cyclic nucleotide-gated channels and T-type Ca2^+^ channels [44]. From here we postulated REST-Nav1.5/nNav1.5 interrelation in breast cancer. With extensive reports demonstrating REST epigenetic regulation is dependent on its ability to recruit HDACs for transcriptional repression activity [45], therefore, higher mRNA expression level of HDAC1, HDAC2, and HDAC3 in MCF-7 were expected. Recent study showed that HDAC1 was highly expressed in hormone receptor positive breast tumor [34] which linked to MCF-7 cells, a human breast cancer cell line widely used for in vitro model with positive estrogen receptor. Our postulation extended to REST-HDAC-Nav1.5/nNav1.5 interrelation in breast cancer.

Studies have shown HDAC inhibitors are able to restore the expression of VGSC isoforms in certain pathological conditions arisen from abnormally repression of VGSCs. For example, TSA was demonstrated to re-express Nav1.8 protein expression in dorsal root ganglion of nerve injury-induced neuropathic pain [46]. Similarly, with another famously known HDAC inhibitor, suberoylanilide hydroxamic acid (SAHA), re-expression of Nav1.5 protein expression was achieved in Duchenne muscular dystrophic mice model which lack Nav1.5 [47]. Additionally, with another known HDAC inhibitor, valproic acids (VPA), chronic treatment with VPA up-regulates the mRNA and cell surface expression of Nav1.7 in adrenal chromaffin cells [48]. Herein, TSA treatment resulted in a significant increase of Nav1.5 and nNav1.5 mRNA expression level in MCF-7 cells (p < 0.01 at 10 000 ng/ml TSA). Subsequently, the effect of TSA on HDACs and REST gene expression were measured to ensure that the observed changes in Nav1.5 and nNav1.5 expression stemmed from REST/HDACs targeting. Indeed, mRNA expression level of REST, HDAC1 and HDAC2 in MCF-7 cells was downregulated by TSA (though only effect on REST and HDAC2 was statistically significant, p < 0.05).

In normal epithelial cells, REST is expressed abundantly and functions as tumor suppressor [49] where lack of REST have been implicated in carcinomas of breast [50], colorectal [27], and small cell lung [51]. In fact, REST is shown to be lost in breast tissues of patients with aggressive phenotype (significant poor prognosis and more than twice as likely to undergo disease recurrence within the first 3 years after diagnosis) [52]. The significantly lower REST expression detected in aggressive Nav1.5/nNav1.5-expressing MDA-MB-231 cells compared to less aggressive MCF-7 and REST downregulation followed by up-regulation of Nav1.5/nNav1.5 after TSA treatment in MCF-7 cells support our hypothesis of REST interrelation with Nav1.5/nNav1.5.

Meanwhile, reports demonstrated that HDAC1 and HDAC2 have a high degree of homology and are able to form a complex in the nucleus [45, 46] which explain the similar effects obtained by TSA on both molecules in MCF-7 cells. In our model of less aggressive breast cancer, the significant reduction of HDAC2 expression was in accordance to colon cancer where HDAC2 was reported to share similar feature to those of tumor-suppressor genes when mutation-loss of HDAC2 function lead to oncogenesis [53].

On the contrary, HDAC3 expression was significantly increased after TSA treatment (p < 0.05). The increased pattern of HDAC3 gene expression level by TSA in MCF-7 cells was similar to that reported by Duong et al. [54] using the similar cell line. Opposite to HDAC1/HDAC2, overexpression of HDAC3 in breast cancer is associated with clinicopathological indicator of disease progression [34], which is in line with our result , HDAC3 increased of expression after treatment with TSA in MCF-7. The role of HDAC3 as tumour suppressor gene is only recognisable in hepatocellular carcinomas, where previously, Bhaskara et al. [55] observed that liver-specific HDAC3 knockdown resulted in overt hepatocellular carcinomas. Thus, our results support the role of HDAC2 as another epigenetic regulator for Nav1.5 and nNav1.5 expression in breast cancer.

TSA was first recognized for its anticancer activity via cell cycle arrest and apoptosis in several types of cancer including breast cancer [51, 52]. The drug has been reported to induce caspase activity and apoptosis in MCF-7 cells via a cytochrome c-dependent pathway [56]. Unfortunately, in clinical experiments, HDAC inhibitors including TSA seem to have serious limitations which hindered their therapeutic potential. For example, the current two structurally distinct HDAC inhibitors – SAHA (vorinostat, Zolinza™) and FK228 (romidepsin, Istodax™), both are reported to have not been effective in clinical trials involving solid tumors i.e. refractory breast, colorectal, non-small cell lung and thyroid cancers [57] and most importantly, these drugs cause serious cardiac toxicity [58].

In this study, despite of growth inhibition by TSA, remarkably, motility and migration (p < 0.05) of MCF-7 cells were still significantly increased. These findings were in line with several other studies which demonstrated similar enhancement of metastatic parameters e.g. invasion and migration by TSA in various human cancers in vitro including those of neuroblastoma, meningioma, and prostate [59], rhabdomyosarcoma [60], breast, gastric, liver, and lung cancer cell lines [61]. In fact, in vivo, treatment with TSA also significantly promotes metastasis in nude mice [61]. Subsequently, the underlying mechanism of metastatic enhancement by TSA was reported to be due to up-regulation of metastatic related markers e.g. urokinase plasminogen activator [59], PKCs [61] and Ezrin [60]. Additionally, TSA also led to prostate cancer aggressiveness via induction of epithelial-to-mesenchymal transition phenotype which associated with increased expression of transcription factors ZEB1, ZEB2 and Slug, and mesenchymal markers such as vimentin, N-cadherin and fibronectin [62]. As in the present study, enhancement of motility and migration of MCF-7 cells by TSA were likely due to the increased expression level of Nav1.5 and nNav1.5. Both Nav1.5 and nNav1.5 are already recognised as a potent metastatic gene in potentiating breast cancer metastatic parameters i.e. motility, migration and invasion when specific VGSC blocker, TTX and other VGSC blockers e.g. phenytoin, ranolazine have all precisely suppressed breast cancer metastasis in vitro and in vivo even at dose concentration that does not interfere with proliferation [5, 13, 63–66]. Enhanced motility and migration of MCF-7 cells after TSA treatment were also pivotal to support the functional re-expression of Nav1.5/nNav1.5. Importantly, our findings could explicate cellular mechanisms for the previous disappointments in clinical experience with HDAC inhibitors in patients with solid tumors.

## Conclusions

Overall, our study demonstrated that epigenetics play role in controlling Nav1.5/nNav1.5 expression breast cancer. Downregulation of REST and HDAC2 expression level by TSA lead to enhanced Nav1.5 and nNav1.5 expression that transformed the less aggressive, MCF-7 cells to gain aggressiveness. We postulated that when REST and HDAC2 are lacking in breast cancer, enhance expression of Nav1.5 and nNav1.5 promotes aggressiveness.

## Abbreviations

VGSCs: voltage-gated sodium channels
HDACs: histone deacetylases
TSA: trichostatin A
HDACis: histone deacetylase inhibitors
Nav1.5: voltage-gated sodium channel 1.5
nNav1.5: neonatal splice variant of Nav1.5
HDAC1: histone deacetylase 1
HDAC2: histone deacetylase 2
HDAC3: histone deacetylase 3
MMP2: matrix metalloprteinase 2

## References

[1] Catterall WA (2000). From ionic currents to molecular mechanisms: the structure and function of voltage-gated sodium channels. Neuron.

[2] Grimes JA, Fraser SP, Stephens GJ, Downing JE, Laniado ME, Foster CS (1995). Differential expression of voltage-activated Na+ currents in two prostatic tumour cell lines: contribution to invasiveness in vitro. FEBS Lett.

[3] Bennett ES, Smith BA, Harper JM (2004). Voltage-gated Na+ channels confer invasive properties on human prostate cancer cells. Eur J Physiol.

[4] Roger S, Besson P, Le Guennec JY (2003). Involvement of a novel fast inward sodium current in the invasion capacity of a breast cancer cell line. Biochim Biophys Acta Biomembr.

[5] Fraser SP, Diss JKJ, Chioni A-M, Mycielska ME, Pan H, Yamaci RF (2005). Voltage-gated sodium channel expression and potentiation of human breast cancer metastasis. Clin Cancer Res.

[6] Onganer PU, Djamgoz MBA (2005). Small-cell lung cancer (human): potentiation of endocytic membrane activity by voltage-gated Na+ channel expression in vitro. J Membr Biol.

[7] Fulgenzi G, Graciotti L, Faronato M, Virginia M, Miceli F, Amoroso S (2006). Human neoplastic mesothelial cells express voltage-gated sodium channels involved in cell motility. Int J Biochem Cell Biol.

[8] Roger S, Rollin J, Barascu A, Besson P, Raynal P-I, Iochmann S (2007). Voltage-gated sodium channels potentiate the invasive capacities of human non-small-cell lung cancer cell lines. Int J Biochem Cell Biol.

[9] Diaz D, Delgadillo DM, Herna E, Mari LUZ, Hinojosa A, Ortiz CS (2007). Functional expression of voltage-gated sodium channels in primary cultures of human cervical cancer. J Cell Physiol.

[10] Gao R, Shen Y, Cai J, Lei M, Wang Z (2010). Expression of voltage-gated sodium channel a subunit in human ovarian cancer. Oncol Rep.

[11] House CD, Vaske CJ, Schwartz AM, Obias V, Frank B, Luu T (2010). Voltage-gated Na+ channel SCN5A is a key regulator of a gene transcriptional network that controls colon cancer invasion. Cancer Res.

[12] Yang M, Kozminski DJ, Wold LA, Modak R, Calhoun JD, Isom LL (2012). Therapeutic potential for phenytoin: targeting Nav1.5 sodium channels to reduce migration and invasion in metastatic breast cancer. Breast Cancer Res Treat.

[13] Nelson M, Yang M, Dowle AA, Thomas JR, Brackenbury WJ (2015). The sodium channel-blocking antiepileptic drug phenytoin inhibits breast tumour growth and metastasis. Mol Cancer.

[14] Brackenbury WJ, Djamgoz MBA (2007). Nerve growth factor enhances voltage-gated Na þ channel activity and transwell migration in Mat-LyLu rat prostate cancer cell line. J Cell Physiol.

[15] Onganer PU, Djamgoz MB (2007). Epidermal growth factor potentiates in vitro metastatic behaviour of human prostate cancer PC-3M cells: involvement of voltage-gated sodium channel. Mol Cancer.

[16] Fraser SP, Ozerlat-Gunduz I, Onkal R, Diss JKJ, Latchman DS, Djamgoz MB (2007). Estrogen and non-genomic upregulation of voltage-gated Na(+) channel activity in MDA-MB-231 human breast cancer cells: role in adhesion. J Cell Physiol.

[17] Brackenbury WJ, Djamgoz MB (2006). Activity-dependent regulation of voltage-gated Na+ channel expression in Mat-LyLu rat prostate cancer cell line. J Physiol.

[18] Diss JKJ, Fraser SP, Walker MM, Patel A, Latchman DS, Djamgoz MB (2008). Beta-subunits of voltage-gated sodium channels in human prostate cancer: quantitative in vitro and in vivo analyses of mRNA expression. Prostate Cancer Prostatic Dis.

[19] Sharma S, Kelly TK, Jones PA (2010). Epigenetics in cancer. Carcinogenesis.

[20] Fraser SP, Ozerlat-Gunduz I, Brackenbury WJ, Fitzgerald EM, Campbell TM, Coombes RC (2014). Regulation of voltage-gated sodium channel expression in cancer: hormones, growth factors and auto-regulation. Philos Trans R Soc Lond B Biol Sci.

[21] Mori N, Schoenherr C, Vandenbergh DJ, Anderson DJ (1992). A common silencer element in the SCG10 and type II Na+ channel genes binds a factor present in nonneuronal cells but not in neuronal cells. Neuron.

[22] Chong JA, Tapia-Ramírez J, Kim S, Toledo-Aral JJ, Zheng Y, Boutros MC (1995). REST: a mammalian silencer protein that restricts sodium channel gene expression to neurons. Cell.

[23] Schoenherr CJ, Paquette AJ, Anderson DJ (1996). Identification of potential target genes for the neuron-restrictive silencer factor. Proc Natl Acad Sci USA.

[24] Ballas N, Battaglioli E, Atouf F, Andres ME, Chenoweth J, Anderson ME (2001). Regulation of neuronal traits by a novel transcriptional complex. Neuron.

[25] Roopra A, Sharling L, Wood IC, Briggs T, Bachfischer U, Paquette AJ (2000). Transcriptional repression by neuron-restrictive silencer factor is mediated via the Sin3-histone deacetylase complex. Mol Cell Biol.

[26] Van Swearingen AE, Siegel MB, Anders CK (2014). Breast cancer brain metastases: evidence for neuronal-like adaptation in a “breast-to-brain” transition?. Breast Cancer Res.

[27] Westbrook TF, Martin ES, Schlabach MR, Leng Y, Liang AC, Feng B (2005). A genetic screen for candidate tumor suppressors identifies REST. Cell.

[28] Robey RW, Chakraborty AR, Basseville A, Luchenko V, Zhan Z, Bates SE (2011). Histone deacetylase inhibitors: emerging mechanisms of resistance. Mol Pharm.

[29] Mottamal M, Zheng S, Huang TL, Wang G (2015). Histone deacetylase inhibitors in clinical studies as templates for new anticancer agents. Molecules.

[30] Moradzadeh M, Tabarraei A, Sadeghnia HR (2015). The role of histone deacetylase (HDAC) as a biomarker in cancer. Mol Biomark Diagn.

[31] Mutze K, Langer R, Becker K, Ott K, Novotny A, Luber B (2010). Histone deacetylase (HDAC) 1 and 2 expression and chemotherapy in gastric cancer. Ann Surg Oncol.

[32] Wu LM, Yang Z, Zhou L, Zhang F, Xie HY, Feng XW (2010). Identification of histone deacetylase 3 as a biomarker for tumor recurrence following liver transplantation in HBV-associated hepatocellular carcinoma. PLoS ONE.

[33] Wang L, Zou X, Berger AD, Twiss C, Peng Y, Li Y (2009). Increased expression of histone deacetylaces (HDACs) and inhibition of prostate cancer growth and invasion by HDAC inhibitor SAHA. Am J Transl Res.

[34] Müller BM, Jana L, Kasajima A, Lehmann A, Prinzler J, Budczies J (2013). Differential expression of histone deacetylases HDAC1, 2 and 3 in human breast cancer–overexpression of HDAC2 and HDAC3 is associated with clinicopathological indicators of disease progression. BMC Cancer.

[35] Ramakrishnan S, Ku S, Ciamporcero E, Miles KM, Attwood K, Chintala S (2016). HDAC 1 and 6 modulate cell invasion and migration in clear cell renal cell carcinoma. BMC Cancer.

[36] Hayashi A, Horiuchi A, Kikuchi N, Hayashi T, Fuseya C, Suzuki A (2010). Type-specific roles of histone deacetylase (HDAC) overexpression in ovarian carcinoma: HDAC1 enhances cell proliferation and HDAC3 stimulates cell migration with downregulation of E-cadherin. Int J Cancer.

[37] Colon-Diaz M, Baez-Vega P, Garcia M, Ruiz A, Monteiro JB, Fourquet J (2012). HDAC1 and HDAC2 are differentially expressed in endometriosis. Reprod Sci.

[38] Livak KJ, Schmittgen TD (2001). Analysis of relative gene expression data using real-time quantitative PCR and the 2^−ΔΔCT^ method. Methods.

[39] Fraser SP, Salvador V, Manning EA, Mizal J, Altun S, Raza M (2003). Contribution of functional voltage-gated Na+ channel expression to cell behaviors involved in the metastatic cascade in rat prostate cancer: I. Lateral motility. J Cell Physiol.

[40] Nadeau H, Lester HA (2002). NRSF causes cAMP-sensitive suppression of sodium current in cultured hippocampal neurons. J Neurophysiol.

[41] Drews VL, Shi K, De Haan G, Meisler MH (2007). Identification of evolutionarily conserved, functional noncoding elements in the promoter region of the sodium channel gene SCN8A. Mamm Genome.

[42] Pozzi D, Lignani G, Ferrea E, Contestabile A, Paonessa F, D’Alessandro R (2013). REST/NRSF-mediated intrinsic homeostasis protects neuronal networks from hyperexcitability. EMBO J.

[43] Chioni AM, Shao D, Grose R, Djamgoz MBA (2010). Protein kinase A and regulation of neonatal Nav1.5 expression in human breast cancer cells: activity-dependent positive feedback and cellular migration. Int J Biochem Cell Biol.

[44] Kuwahara K (2013). Role of NRSF/REST in the regulation of cardiac gene expression and function. Circ J.

[45] Paonessa F, Criscuolo S, Sacchetti S, Amoroso D, Scarongella H, Pecoraro Bisogni F (2016). Regulation of neural gene transcription by optogenetic inhibition of the RE1-silencing transcription factor. Proc Natl Acad Sci USA.

[46] Matsushita Y, Araki K, Omotuyi OI, Mukae T, Ueda H (2013). HDAC inhibitors restore C-fibre sensitivity in experimental neuropathic pain model. Br J Pharmacol.

[47] Colussi C, Berni R, Rosati J, Straino S, Vitale S, Spallotta F (2010). The histone deacetylase inhibitor suberoylanilide hydroxamic acid reduces cardiac arrhythmias in dystrophic mice. Cardiovasc Res.

[48] Yamamoto R, Yanagita T, Kobayashi H, Yokoo H, Wada A (1997). Up-regulation of sodium channel subunit mRNAs and their cell surface expression by antiepileptic valproic acid: activation of calcium channel and catecholamine secretion in adrenal chromaffin cells. J Neurochem.

[49] Reddy BY, Greco SJ, Patel PS, Trzaska KA, Rameshwar P (2009). RE-1-silencing transcription factor shows tumor-suppressor functions and negatively regulates the oncogenic TAC1 in breast cancer cells. Proc Natl Acad Sci USA.

[50] Negrini S, Prada I, D’Alessandro R, Meldolesi J (2013). REST: an oncogene or a tumor suppressor?. Trends Cell Biol.

[51] Coulson JM, Edgson JL, Woll PJ, Quinn JP (2000). A splice variant of the neuron-restrictive silencer factor repressor is expressed in small cell lung cancer: a potential role in derepression of neuroendocrine genes and a useful clinical marker. Cancer Res.

[52] Wagoner MP, Gunsalus KTW, Schoenike B, Richardson AL, Friedl A, Roopra A (2010). The transcription factor REST is lost in aggressive breast cancer. PLoS Genet.

[53] Ropero S, Fraga MF, Ballestar E, Hamelin R, Yamamoto H, Boix-Chornet M (2006). A truncating mutation of HDAC2 in human cancers confers resistance to histone deacetylase inhibition. Nat Genet.

[54] Duong V, Bret C, Altucci L, Mai A, Duraffourd C, Loubersac J (2008). Specific activity of class II histone deacetylases in human breast cancer cells. Mol Cancer Res.

[55] Bhaskara S, Knutson SK, Jiang G, Chandrasekharan MB, Wilson AJ, Zheng S (2010). Hdac3 is essential for the maintenance of chromatin structure and genome stability. Cancer Cell.

[56] Medina V, Edmonds B, Young GP, James R, Appleton S, Zalewski PD (1997). Induction of caspase-3 protease activity and apoptosis by butyrate and trichostatin a (inhibitors of histone deacetylase): dependence on protein synthesis and synergy with a mitochondrial/cytochrome c-dependent pathway. Cancer Res.

[57] Vansteenkiste J, Van Cutsem E, Dumez H, Chen C, Ricker JL, Randolph SS (2008). Early phase II trial of oral vorinostat in relapsed or refractory breast, colorectal, or non-small cell lung cancer. Invest New Drugs.

[58] Gryder BE, Sodji QH, Oyelere AK (2013). Targeted cancer therapy: giving histone deacetylase inhibitors all they need to succeed. Future Med Chem.

[59] Pulukuri SMK, Gorantla B, Rao JS (2007). Inhibition of histone deacetylase activity promotes invasion of human cancer cells through activation of urokinase plasminogen activator. J Biol Chem.

[60] Yu Y, Zeng P, Xiong J, Liu Z, Berger SL, Merlino G (2010). Epigenetic drugs can stimulate metastasis through enhanced expression of the pro-metastatic Ezrin gene. PLoS ONE.

[61] Lin KT, Wang YW, Chen CT, Ho CM, Su WH, Jou YS (2012). HDAC inhibitors augmented cell migration and metastasis through induction of PKCs leading to identification of low toxicity modalities for combination cancer therapy. Clin Cancer Res.

[62] Kong D, Ahmad A, Bao B, Li Y, Banerjee S, Sarkar FH (2012). Histone deacetylase inhibitors induce epithelial-to-mesenchymal transition in prostate cancer cells. PLoS ONE.

[63] Brackenbury WJ, Chioni AM, Diss JKJ (2007). Djamgoz MBA. The neonatal splice variant of Nav1.5 potentiates in vitro invasive behaviour of MDA-MB-231 human breast cancer cells. Breast Cancer Res Treat.

[64] Driffort V, Gillet L, Bon E, Marionneau-Lambot S, Oullier T, Joulin V (2014). Ranolazine inhibits NaV1.5-mediated breast cancer cell invasiveness and lung colonization. Mol Cancer.

[65] Martin F, Ufodiama C, Watt I, Bland M, Brackenbury WJ (2015). Therapeutic value of voltage-gated sodium channel inhibitors in breast, colorectal, and prostate cancer: a systematic review. Front Pharmacol.

[66] Mohammed FH, Khajah MA, Yang M, Brackenbury WJ, Luqmani YA (2016). Blockade of voltage-gated sodium channels inhibits invasion of endocrine-resistant breast cancer cells. Int J Oncol.

